# A positive feedback loop between ID3 and PPARγ via DNA damage repair regulates the efficacy of radiotherapy for rectal cancer

**DOI:** 10.1186/s12885-023-10874-7

**Published:** 2023-05-11

**Authors:** Chuanzhong Huang, Ling Wang, Huijing Chen, Wankai Fu, Lingdong Shao, Dongmei Zhou, Junxin Wu, Yunbin Ye

**Affiliations:** 1 Laboratory of Immuno-Oncology, Clinical Oncology School of Fujian Medical University, Fujian Cancer Hospital, Fuzhou, 350014 China; 2 School of Basic Medical Sciences, Fujian Medical University, Fuzhou, 350122 China; 3 Fujian Provincial Key Laboratory of Translational Cancer Medicine, Fuzhou, 350014 China; 4 Department of Radiation Oncology, Clinical Oncology School of Fujian Medical University, Fujian Cancer Hospital, Fuzhou, 350014 China; 5 Departments of Pathology, Clinical Oncology School of Fujian Medical University, Fujian Cancer Hospital, Fuzhou, 350014 China

**Keywords:** ID3, PPARγ, Radiotherapy efficacy, Positive feedback, Rectal cancer

## Abstract

**Objective:**

To study the effect of inhibitor of differentiation 3 (ID3) on radiotherapy in patients with rectal cancer and to explore its primary mechanism.

**Methods:**

Cell proliferation and clonogenic assays were used to study the relationship between ID3 and radiosensitivity. Co-immunoprecipitation and immunofluorescence were performed to analyze the possible mechanism of ID3 in the radiosensitivity of colorectal cancer. At the same time, a xenograft tumor model of HCT116 cells in nude mice was established to study the effect of irradiation on the tumorigenesis of *ID3* knockdown colorectal cancer cells in vivo. Immunohistochemistry was performed to analyze the relationship between ID3 expression and the efficacy of radiotherapy in 46 patients with rectal cancer.

**Results:**

Proliferation and clonogenic assays revealed that the radiosensitivity of colorectal cancer cells decreased with *ID3* depletion through p53–independent pathway. With the decrease in *ID3* expression, MDC1 was downregulated. Furthermore, the expression of ID3, MDC1, and γH2AX increased and formed foci after irradiation. ID3 interacted with PPARγ and form a positive feedback loop to enhance the effect of ID3 on the radiosensitivity of colorectal cancer. Irradiation tests in nude mice also confirmed that HCT116 cells with *ID3* knockdown were more affected by irradiation. Immunohistochemical study showed that rectal cancer patients with low expression of ID3 had better radiotherapy efficacy.

**Conclusions:**

ID3 and PPARγ influence the radiosensitivity of colorectal cancer cells by interacting with MDC1 to form a positive feedback loop that promotes DNA damage repair. Patients with low expression of ID3 who received neoadjuvant chemoradiotherapy can obtain a better curative effect.

**Supplementary Information:**

The online version contains supplementary material available at 10.1186/s12885-023-10874-7.

## Highlights

• Depletion of ID3 enhances radiosensitivity of rectal cancer cells.

• Interaction between ID3 and MDC1 promotes DNA damage repair.

• Positive feedback loop between PPARγ and ID3 enhances the radiosensitivity of rectal cancer cells.

• Patients with low expression of ID3 can obtain a better radiotherapy effect.

**Supplementary Information:**

The online version contains supplementary material available at 10.1186/s12885-023-10874-7.

## Introduction

Rectal cancer is one of the major diseases to endanger human health. Globally, the incidence rate of rectal cancer is 3.9% among all new cancer cases, and the mortality rate is 3.2% among all deaths from cancer [1], both ranking eighth worldwide. Surgical treatment remains the main choice for rectal cancer patients to obtain a radical cure, but even after surgery and comprehensive treatment, the local recurrence rate and distant metastasis rate can be as high as 30% or more [2].

Radiotherapy is an important mode of tumor treatment. Many studies have shown that preoperative neoadjuvant chemoradiotherapy (NCRT) can downstage tumors, increase the rate of surgical resection, reduce the local recurrence rate, and enable disease-free progression in advanced rectal cancer [3, 4]. At present, patients with advanced rectal cancer receive preoperative adjuvant radiotherapy as the European standard [5]. However, there are great individual differences in the therapeutic effects of preoperative neoadjuvant chemoradiotherapy, and approximately 20%–40% of patients are insensitive to it [6]. In-depth studies of the molecular mechanism of rectal cancer radiosensitivity and identification of biomarkers related to radiosensitivity will be of important clinical significance for the individualized treatment of rectal cancer, to allow screening of patients to avoid ineffective and excessive treatment or enable appropriate intervention for effective targets to improve the radiosensitivity of tumors.

Inhibitor of differentiation (ID), also known as inhibitor of DNA binding, plays a very important role in cell differentiation, proliferation, tumorigenesis, invasion, angiogenesis, and anti-apoptosis [7]. It has four members, ID1, ID2, ID3, and ID4, which all belong to the helix–loop–helix transcription factor family [8]. Because of the lack of DNA binding domain, ID proteins form a non-functional heterodimer after binding with basic helix–loop–helix (bHLH) transcription factors such as E protein, which inhibits the transcriptional activity of bHLH, thus preventing cell differentiation and promoting cell proliferation [9]. An increasing number of studies has found that ID3 may play a more important role in tumorigenesis and development [10]. ID3 can be induced by calcium-binding protein S100A8 and inhibits p21 to regulate the cell cycle and proliferation of colorectal cancer cells [11]. ID3 is also associated with chemoresistance, and depletion of ID3 increases the sensitivity of melanoma to short-term treatment with the BRAF inhibitor vemurafenib. Thus, it may be a new critical molecule of adaptive resistance and a potential drug target [12]. However, the effect of ID3 on the biological characteristics of rectal cancer and resistance to radiotherapy are rarely reported.

This study aimed to investigate the relationship between ID3 and radiosensitivity in colorectal cancer cell lines through in vivo and in vitro experiments. We explored the effect of ID3 expression on the efficacy of radiotherapy in patients with rectal cancer, examined its clinical application value, and analyzed its primary mechanism.

## Materials and methods

### Materials and cell lines

Fetal bovine serum, McCoy's 5A culture medium, trypsin and puromycin were purchased from Gibco (Grand Island, NY, USA). HCT116 and HT-29 human colorectal cancer cell lines were purchased from the Cell Bank of the Chinese Academy of Sciences (Shanghai, China). All antibodies used in the experiments were purchased from Cell Signaling Technology (Danvers, MA, USA), except for ID3 antibody, which was purchased from Sigma–Aldrich (Saint Louis, MO, USA). ID3 overexpression plasmid and control plasmid (pcDNA3.1-3flag-ZsGreen-Puro), and *ID3* siRNA and its control siRNA were provided by Hanbio Biotechnology Co. Ltd. (Shanghai, China). All chemicals were of analytical reagent grade. Water for all reactions, solution preparation, and sample purification was double-distilled.

Paraffin specimens for immunohistochemical were pre-irradiation biopsy tissues without any treatment from 46 patients who received preoperative neoadjuvant chemoradiotherapy at Fujian Cancer Hospital from 2010 to 2020 and were diagnosed with rectal cancer by clinicopathology. Other inclusion criteria were: 1) surgery was followed by radiotherapy with tumor regression grade data; 2) no previous history of cancers at other sites or other concomitant malignant diseases. Patients who were included in blind treatment in other clinical trials were screened out. The group included 25 men and 21 women with a mean age of 53.5 years and a mean age of 52 years (Table 1).

**Table 1:** Characteristics of patients

Characteristics	N(%)
Median Age, year (range)	53.5(30–81)
≤ 50	18(39.1%)
> 50	28(60.9%)
Gender	
Male	25(54.3%)
Female	21(45.7%)
T classification	
1	1(2.2%)
2	8(17.4%)
3	26(56.5%)
4	11(23.9%)
N classification	
0	22(47.8%)
1	16(34.8%)
2	8(17.4%)
3	0(0.0%)
M classification	
x	0(0.0%)
0	42(91.3%)
1	4(8.7%)
Clinical stage	
I	5(10.9%)
II	15(32.6%)
III	22(47.8%)
IV	4(8.7%)
Preoperative radiotherapy	
25 Gy	17(100.0%)
50 Gy	29(0.0%)
Preoperative chemotherapy	
Yes	36(78.3%)
Induction	8(17.4%)
Concurrent	24(52.2%)
Induction + Concurrent	4(8.7%)
No	10(21.7%)
Chemotherapy cycles	
≤ 3	30(65.2%)
> 3	16(34.8%)

### Cell culture

HCT116 and HT-29 colorectal cancer cells were cultured in McCoy's 5A medium supplemented with 10% fetal bovine serum at 37 °C in 5% carbon dioxide. The cells were cultured to approximately 70% confluency in complete medium for transfection and western blot experiments, and approximately 30% confluency for clonogenic, cell proliferation assay, and immunofluorescence assay.

### Plasmid constructs and transfection

Lipofectamine 3000 was used to transfect plasmids and siRNA into HCT116 and HT-29 cells. The cells transfected with empty plasmid were HCT116-PC/HT-29-PC and the ID3-overexpress plasmid were HCT116-ID3 OE/HT-29-ID3 OE. The cells transfected with control siRNA were HCT116-NC/HT-29-NC and the siID3 were HCT116-ID3 KD/HT-29-ID3 KD. The expression of ID3 was detected by western blotting. Stable *ID3* knockdown HCT116 and HT-29 were screened with puromycin at 1.5 μg/ml (Sigma) for at least 1 week for nude mice experiments.

### Western blot assay

HCT116 and HT-29 were centrifuged and harvested after trypsin digestion. The cells were lysed and the pelleted proteins were quantified by BCA assay. The protein samples were electrophoresed on 12% SDS-PAGE gel for 2–3 h. Proteins from the gels were transferred to nitrocellulose(NC) filter membranes for 1 h at 60 V in transfer buffer (48 mM Tris, 39 mM glycine, and 20% methanol) at 4 °C. After the membranes were blocked, they were incubated overnight at 4 °C with primary antibody, and then rinsed and incubated with horseradish peroxidase-conjugated secondary antibody. Results were visualized with SuperSignal West Pico kit (Thermo Fisher Scientific, USA) using Chemiluminescence Apparatus (Bio-Rad, USA). The primary antibodies used in this study were anti-ID3 (1:500), anti-MDC1 (1:500), anti-γH2AX(1:500), anti-PPARγ (1:1000), anti-p53(1:500), anti-mutant p53(1:500) and anti-β-actin (1:1000). All the original blot images were in Supplementary material. Several blots showed not full length membranes because they were cut prior to hybridisation with antibodies or enlarged as much as possible to obtain the clearest image before photographing.

### X-ray irradiation

HCT116 and HT-29 cells in logarithmic growth stage were digested by trypsin and counted. They were placed in a Petri dish and irradiated vertically by a medical linear accelerator. The irradiation doses were 0, 2, 4, 6 and 8 Gy, the dose rate was 400 MU/min, the energy was 6 m, and the irradiation field was 10 × 10 cm, with 2 cm tissue equivalent filler on the surface during irradiation. Similarly, colorectal cancer cells were irradiated with 6 Gy for 1, 2, and 4 h, and then digested and harvested for western blotting.

### Clonogenic assay

Cell suspensions were diluted according to cell proliferation ability and irradiation dose, then seeded into 12-well plates followed by incubation at 37 °C with 5% CO_2_ for 10–14 days. There were 50 cells/well for the control group, 200 cells/well for the 2 Gy group, 400 cells/well for the 4 Gy group, 1000 cells/well for the 6 Gy group, and 2000 cells/well for the 8 Gy group. Surviving colonies were stained with crystal violet, counted, and then imaged by a Bio-rad GS 800 Optical density scanner. According to the number of inoculations, the cell survival rate was calculated and statistical analysis was conducted. Cell survival rate = (clonogenic rate of irradiated cells/clonogenic rate of control cells) × 100%.

### Cell proliferation assay

Cell proliferation was assessed using the WST method. HCT116 and HT-29 cells irradiated with different doses were seeded into 96-well plates at a density of 5000 cells/well, followed by incubation at 37 °C in an environment with 5% CO_2_ for 72 h. Then 10 μl WST solution was added to each well and incubated at 37 °C for 3 h. The absorbance of each well was determined at 450 nm with a microplate reader. Proliferation inhibition rate = [OD value of control group − OD value of irradiated group)/OD value of control group] × 100%.

### Apoptosis assay

Apoptosis analysis was performed to measure the cell apoptosis after radiation by flow cytometry (FCM). In brief, cells were harvested and then washed with phosphate-buffered saline (PBS) by centrifugation at 1000 × g for 5 min at room temperature. The cells were stained with AnnexinV/PI Apoptosis Detection Kit at 37 °C for 30 min in dark, washed with PBS. And then subjected to FCM. The experiments were performed in triplicate.

### Immunofluorescence

To visualize DNA damage foci and colocalization between ID3 and MDC1, cells cultured on coverslips were irradiated at 6 Gy and cultured at 37 °C for 6 h. After washing twice with PBS and fixing with 4% paraformaldehyde for 10 min, the coverslips were permeabilized with 0.5% Triton X-100 for 20 min at room temperature. After blocking with 5% BSA in TBST, the cells were single- or double-immunostained with primary antibodies at 4 °C overnight, washed with TBST, and then incubated with appropriate Alexa Fluor 488 (green; Molecular Probe)- or Alexa Fluor 594 (red; Molecular Probe)-conjugated secondary antibodies. DAPI was added and the cells were incubated in the dark for 5 min to stain the nuclei. After sealing, fluorescence images were observed by fluorescence microscopy.

### Comet assay

According to OxiSelect™ Comet Assay Kit, heat comet agarose until agarose liquefies. Add 75 µL of agarose per well onto the comet slide to create a base layer. Centrifuge cancer cells and wash them with PBS, resuspend the cells at 1 × 10^5^ cells/mL in ice-cold PBS. Combine cell samples with comet agarose at 1:10 ratio (v/v), mix well by pipetting, and immediately transfer 75 µL/well onto the top of the comet agarose base layer. Carefully, transfer the slide to a small container containing pre-chilled lysis buffer, then replace with pre-chilled alkaline solution. Fill the chamber with cold TBE electrophoresis solution until the buffer level covers the slide. Apply voltage to the chamber at 15 V for 15 min. Immerse the slide in the ddH2O and 70% Ethanol, then allow to air dry. Incubate the slide with 100 µL/well of diluted Vista Green DNA Dye. View slides by epifluorescence microscopy using a FITC filter. The DNA damage is quantified by measuring the displacement between the genetic material. Tail DNA% = 100 × Tail DNA Intensity/Cell DNA Intensity. Tail Moment = Tail DNA% × Tail Moment length.

### Co-immunoprecipitation (co-IP)

HCT116-Id3 OE cells were lysed and some of the supernatant was analyzed by western blot, with the remainder gently mixed overnight at 4 °C with anti-Myc agarose beads and anti-IgG agarose beads as a control. After washing twice with PBS, the immunoprecipitated complexes were analyzed by western blotting.

### In-vivo animal experiments

Specific pathogen-free female BALB/c nude mice aged 4–6 weeks were purchased from Shanghai SLAC Laboratory Animal Co. Ltd. HCT116-NC and HCT116-Id3 KD cells in logarithmic proliferation stage were harvested and suspended in PBS. Each nude mouse was anesthetized with pentobarbital and subcutaneously inoculated with 2 × 10^6^ cells in the foreleg and hind leg. When the tumor size reached approximately 50–60 mm^3^ (tumor volume = length × wide × wide × 0.5), the xenograft tumors on the hind leg were irradiated with 8 Gy every 4 days for a total of four times, while the xenograft tumors on the foreleg were not irradiated. After 2 weeks, the mice were euthanized with carbon dioxide, and the tumor diameters were measured using digital calipers. All animals were maintained under specific pathogen-free conditions and all experiments were performed in accordance with the Animal Care Committee of Fujian Medical University, China.

### Tumor regression grade (TRG) system

Surgical specimens of rectal cancer patients receiving NCRT were fixed in buffered formalin and embedded in paraffin for pathological diagnosis. According to the system recommended by the American Joint Committee on Cancer Staging Manual (AJCC 8^th^ edition), the grade of tumor response to NCRT was classified into four categories [13, 14]: TRG 0 (complete regression), no residual cancer cells; TRG 1 (near-complete regression), single or small groups of cancer cells; TRG 2 (moderate regression), residual cancer with desmoplastic response; and TRG 3 (minimal regression), minimal evidence of tumor response. Patients with TRG 0–1 were considered to have a good response (effective group), while patients with TRG 2–3 were considered to have a poor response (ineffective group) to NCRT.

### Immunohistochemistry

Paraffin specimens for immunohistochemistry were taken from pre-irradiation biopsy tissues of 46 patients who received radiotherapy treatment before surgery at Fujian Cancer Hospital from 2010 to 2020. Patients were divided into two groups according to postoperative TRG. Complete and moderate reactions were classified as the effective group, and mild and adverse reactions were classified as the ineffective group. The study was approved by the ethics committee of Fujian Cancer Hospital (ethics no. sq-2015–034-01). According to the estimated sample quantity, at least 40 specimens were needed. Thus, a total of 46 cases were included in this study.

According to the immunohistochemical kit manual (KIT-9701, Maixin Technology Co., Ltd, China), paraffin sections of tissues from patients with rectal cancer were baked in a 60 °C incubator. After dewaxing and hydration, they were repaired with citric acid antigen repair solution, and then 50 μl reagent A was added to the sections and incubated. After washing, 50 μl reagent B was added and incubated, followed by incubation with anti-ID3 antibody at 4 °C overnight. After adding secondary antibody and reagent D, freshly prepared DAB reagent was added for staining. Stained sections were examined to identify the cellular localization of ID3 immunoreactivity and were scored by two clinicians for both intensity (− , + , +  + , and +  + +) and proportion (0%, 1%–25%, 26%–50%, 51%–75%, and > 75%) of tumor cells stained.

### Statistical analysis

All data analyses were performed using the SPSS 26.0 statistical software package (IBM, Armonk, NY, USA). The non-parametric test of single ordered R*C list was used for immunohistochemical difference analysis (two independent samples), and other data were analyzed with Student’s t-test. The data represent mean ± SD from at least three independent experiments. A value of *P* < 0.05 was considered statistically significant.

## Results

### Depletion of ID3 enhances radiosensitivity of colorectal cancer cells

**Fig. 1:**
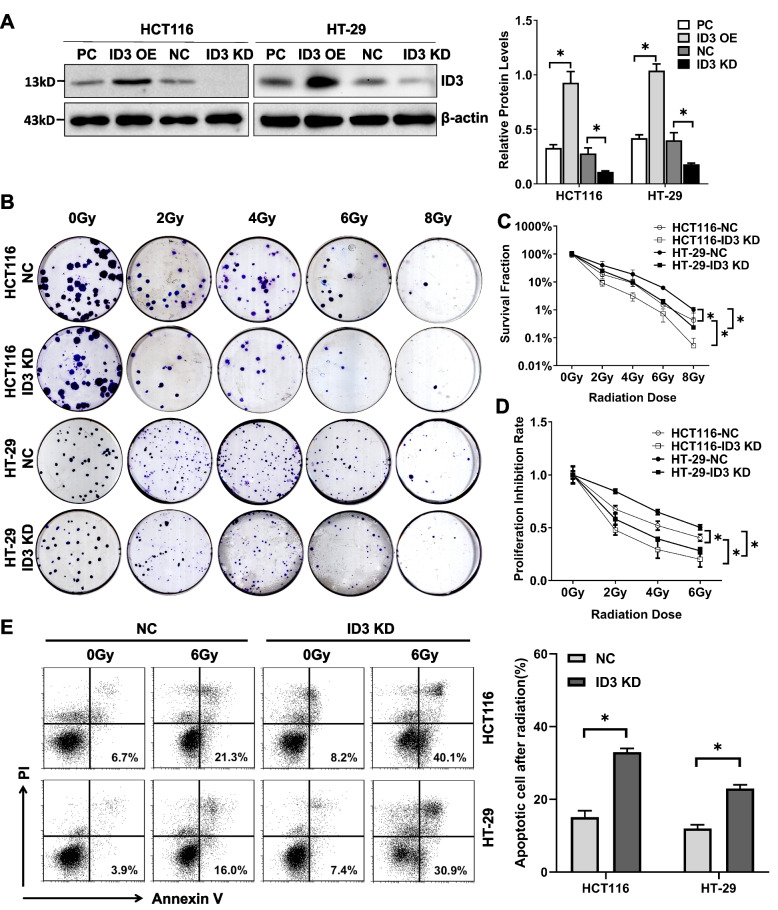
Low expression of ID3 enhanced the radiosensitivity of colorectal cancer cells. **A** Western blotting (left panel) and statistical analysis (right panel) of ID3 protein expression in colorectal cancer cells with ID3 knockdown and overexpress. β-actin was used as a loading control. The expression of ID3 protein decreased significantly in the ID3 KD group and increased significantly in the ID3 OE group. **B** Clonogenic assay to assess the effect of ID3 on the clonogenic activity of colorectal cancer cells after irradiation. **C** Surviving fraction in clonogenic assay. **D** WST assay to assess the effect of ID3 on the proliferation of colorectal cancer cells after irradiation. **E** Flow cytometry assay to assess the effect of ID3 on the apoptosis of colorectal cancer cells after irradiation. Experiments were repeated at least three times. Data are expressed as mean ± SD (*n* = 3). *P < 0.05. NC: siRNA control, PC: pcDNA3.1 control, KD: knockdown, OE: overexpress

To study the effect of ID3 on the radiosensitivity of colorectal cancer cells, we constructed *ID3* knockdown and control cell lines HCT116-NC, HCT116-ID3 KD, HT-29-NC, HT-29-ID3 KD, and ID3 overexpression cell lines and control cell lines HCT116-PC, HCT116-ID3 OE, HT-29-PC, and HT-29-ID3. Western blotting showed that the expression of ID3 protein decreased significantly in the ID3 KD group and increased significantly in the ID3 OE group (Fig. 1A).

Through clonogenic assays, we investigated the changes in sensitivity of HCT116 and HT-29 cells to irradiation after the depletion of ID3 expression. We calculated the cell survival rate to exclude the effect of the change in ID3 expression on cell proliferation. The results showed that HCT116 cells and HT-29 cells had increased radiosensitivity with the depletion of ID3 expression (Fig. 1B and C). Meanwhile, we also verified the results of the clonogenic experiments through cell proliferation assay (WST) and calculated the proliferation inhibition rate to exclude the effect of the change in ID3 expression on proliferation. The results showed that the survival rate of *ID3* knockdown cells decreased significantly and their radiosensitivity increased significantly (Fig. 1D). Furthermore, we analyzed the effect of ID3 on apoptosis of two colon cancer cell lines after radiotherapy. Consistent with the results of clonogenesis and proliferation assays, flow cytometry showed that radiation increased apoptosis of colon cancer cells after ID3 knockdown, suggesting that ID3 knockdown might increase the radiosensitivity of colon cancer cells (Fig. 1E).

**Fig. 2:**
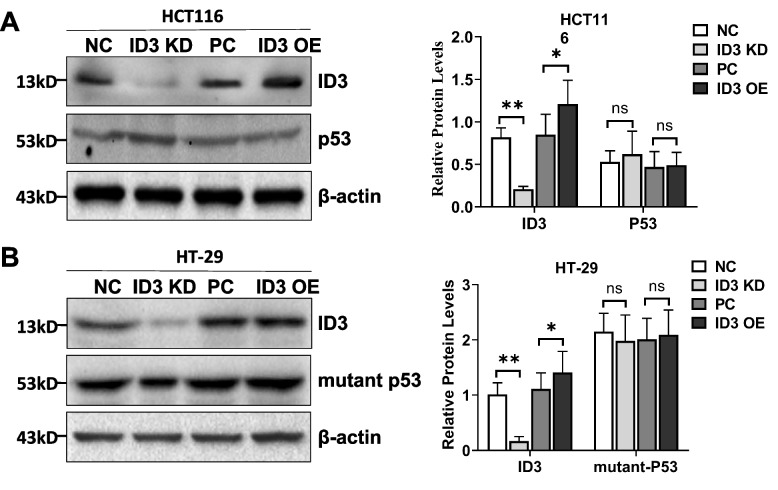
The effect of ID3 on the radiosensitivity of colorectal cancer was through p53–independent pathway. **A** Western blotting (left panel) and statistical analysis (right panel) of ID3 and wild-type p53 protein expression in HCT116 cells. Wild-type p53 expression did not change with ID3. **B** Western blotting (left panel) and statistical analysis (right panel) of ID3 and mutant p53 protein expression in HT-29 cells. Mutant p53 expression did not change with ID3. Experiments were repeated at least three times. Data are expressed as mean ± SD (*n* = 3). *P < 0.05, **P < 0.001. NC: siRNA control, PC: pcDNA3.1 control, KD: knockdown, OE: overexpress

In addition, the p53 signaling pathway plays critical roles in determining the radio-sensitivity of cancer cells [15]. P53 controls a safeguard mechanism that prevents accumulation of abnormal cells and their transformation by regulating DNA repair, cell cycle progression, cell death, or senescence [16]. Is the effect of ID3 on the radiosensitivity of colon cancer cells through p53–dependent or p53–independent mechanisms? Our results had showed that the radiosensitivity of HCT116 cells with wild–type p53 was stronger than that of HT-29 cells with mutant p53. The status of p53 still has an impact on the radiosensitivity. However, HCT116 cells with wild–type p53 and HT-29 cells with mutant p53 had both increased radiosensitivity with the depletion of ID3 expression (Fig. 1C). We also detected the effect of ID3 expression change on wild–type p53 in HCT116 cells and mutant p53 in HT-29 cells. The results showed that ID3 did not affect the expression of wild–type or mutant p53 (Fig. 2A and B). These results showed that ID3 on the radiosensitivity of colorectal cancer was through p53–independent pathway.

### Interaction between ID3 and MDC1 promotes DNA damage repair in colorectal cancer cells

**Fig. 3:**
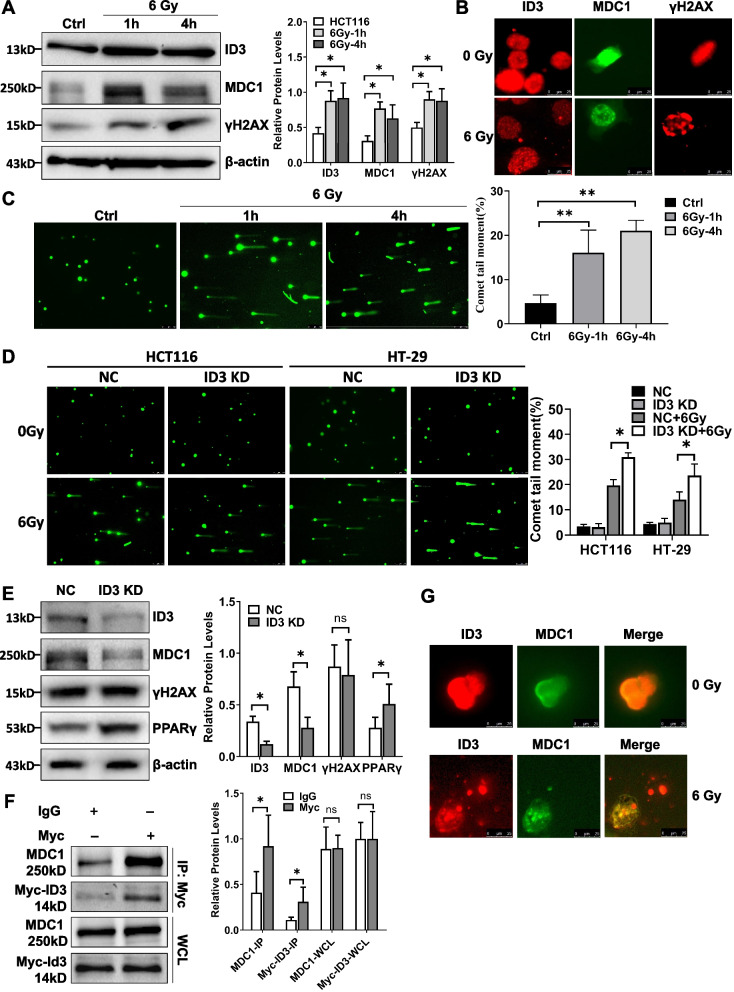
ID3 interacts with MDC1 to promote DDR in colorectal cancer. **A** Western blotting (left panel) and statistical analysis (right panel) of ID3, MDC1 and γH2AX protein expression after irradiation. The protein expression of ID3, MDC1 and γH2AX all increased significantly after irradiation. **B** Immunofluorescence revealed ID3, MDC1, and γH2AX foci formation after irradiation of HCT116 cells. **C** Comet tail moments (left panel) and statistical analysis (right panel) at indicated time points after exposure to IR in HCT116 cells. **D** Comet tail moments (left panel) and statistical analysis (right panel) in both control and ID3-depleted colon cancer cells after exposure to IR. **E** Western blotting (left panel) and statistical analysis (right panel) of ID3, MDC1, γH2AX and PPARγ expression with ID3 knockdown. There was no significant change in γH2AX expression when ID3 expression decreased, but the expression of MDC1 and PPARγ decreased significantly. **F** Lysates of HCT116 cells were subjected to co-immunoprecipitation using an anti-Myc antibody followed by western blotting (left panel) using anti-MDC1 and anti-ID3 antibodies. A statistical graph is located on its right. The result showed that MDC1 interacted with ID3. **G** Immunofluorescence showed ID3 and MDC1 colocalization in HCT116 cells with or without exposure to X-ray irradiation. The cells were exposed to 6 Gy irradiation and fixed at the indicated time points. β-actin was used as a loading control. Experiments were repeated at least three times. Data are expressed as mean ± SD (*n *= 3). *P < 0.05. NC: siRNA control, PC: pcDNA3.1 control, KD: knockdown, OE: overexpress, Ctrl: radiation control

To clarify the specific mechanism of ID3 in reducing the radiosensitivity of colorectal cancer cells, we detected the expression of ID3 and DNA damage repair proteins MDC1 and γH2AX after irradiation by western blotting. The results showed that the protein expression of ID3, MDC1 and γH2AX in HCT116 cells increased significantly after irradiation (Fig. 3A). Meanwhile, immunofluorescence also showed that ID3, MDC1, and γH2AX foci were increased in colorectal cancer cells after irradiation (Fig. 3B). Comet assay also confirmed that DNA damage occurred in HCT116 cells after irradiation (Fig. 3C). After irradiation, the presence of DNA double-strand breaks (DSBs) and the formation of foci indicated that ID3, MDC1, and γH2AX were recruited to the DNA damage site to repair the broken DNA double strand. We next investigated whether ID3 affects DSBs repair by measuring comet tail moments. We found that depletion of ID3 in HCT116 cells had signifificantly more residual DSBs than control cells, as evidenced by the increase in comet tail moments after radiation (Fig. 3D). These results showed that ID3 was upregulated by stress together with DNA damage repair proteins MDC1 and γH2AX and promoted the repair of DSBs.

Next, we investigated the relationship between ID3 and irradiation injury repair-related proteins. Western blotting showed that there was no significant change in γH2AX expression when ID3 expression decreased, but the expression of MDC1 and PPARγ, which can enhance radiosensitivity, decreased significantly (Fig. 3E). Furthermore, co-IP experiments were performed to explore the interaction between ID3 and MDC1. As shown in Fig. 3F, endogenous MDC1 interacted with ID3. In addition, immunofluorescence showed that ID3 and MDC1 were co-localized, and ID3 and MDC1 foci were also co-localized after irradiation (Fig. 3G). These results suggested that ID3 can interact with MDC1 to promote DNA damage repair in colorectal cancer cells.

### Positive feedback loop between PPARγ and ID3 enhances the radiosensitivity of colorectal cancer cells

**Fig. 4:**
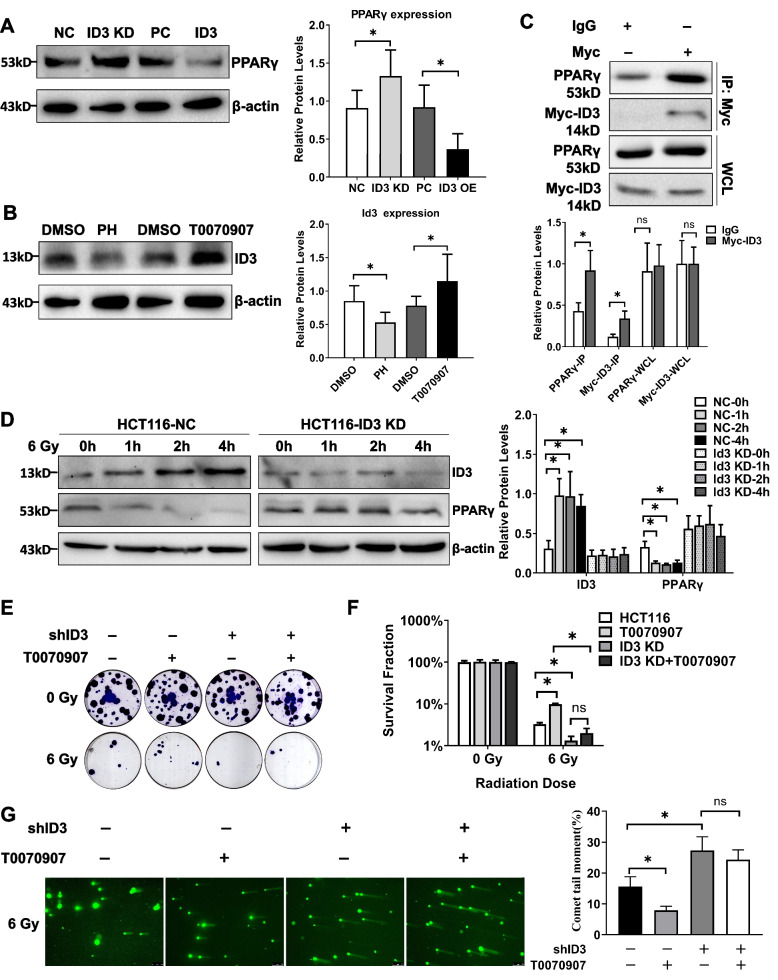
A positive feedback loop between ID3 and PPARγ enhances the radiosensitivity of colorectal cancer cells. **A** Western blotting (left panel) and statistical analysis (right panel) of PPARγ expression in HCT116 cells with ID3 knockdown or overexpression. PPARγ was negatively correlated with the expression of ID3. **B** Western blotting (left panel) and statistical analysis (right panel) of ID3 expression in HCT116 cells treated with PPARγ agonist pioglitazone hydrochloride (PH) or PPARγ inhibitor T0070907. After PPARγ activation, ID3 decreased, while after PPARγ inhibition, ID3 increased. **C** Lysates of HCT116 cells were subjected to co-immunoprecipitation using an anti-Myc antibody followed by western blotting (left panel) using anti-PPARγ and anti-ID3 antibodies. A statistical graph is located on its right. The result showed that MDC1 interacted with ID3. **D** Western blotting (left panel) and statistical analysis (right panel) of ID3 and PPARγ expression in HCT116-NC and HCT116-Id3 KD cells after irradiation. β-actin was used as a loading control. After ID3 depletion, irradiation could no longer affect PPARγ expression. **E** Clonogenic assay was used to assess the effect of ID3 depletion and PPARγ inhibitor T0070907 on the clonogenic activity of HCT116 cells after irradiation. **F** Statistical analysis of the surviving fraction in clonogenic assays. **G**. Comet tail moments in both control and ID3-depleted HCT116 cells after exposure to T0070907 and 6-Gy irradiation. Experiments were repeated at least three times. Data are expressed as mean ± SD (*n* = 3). ∗ *P* < 0.05.NC: siRNA control, PC: pcDNA3.1 control, KD: knockdown, OE: overexpress

Peroxisome proliferators-activated receptor PPARγ not only plays an important role in fat metabolism, but also a significant role in regulating the radiosensitivity of colorectal cancer cells [17]. We investigated the protein levels of PPARγ after *ID3* knockdown and overexpression by western blotting and found that ID3 could inhibit the expression of PPARγ (Fig. 4A). Similarly, after treatment with PPARγ agonist pioglitazone hydrochloride and inhibitor T0070907, the protein level of ID3 in HCT116 cells was opposite to that of PPARγ (Fig. 4B). These results showed that ID3 and PPAR form a contrary positive feedback regulatory circuit. When ID3 is increased, it inhibits PPARγ protein, and when PPARγ is inhibited by ID3, it further promotes ID3 expression, to gradually enhance the malignancy of colorectal cancer cells and radiotherapy resistance.

Next, we investigated the interaction between ID3 and PPARγ by co-IP. The results showed that endogenous PPARγ also interacted with ID3 (Fig. 4C).

Furthermore, we examined the effect of irradiation on the expression of ID3 and PPARγ by western blotting. The results showed that after irradiation, ID3 protein in HCT116 cells increased significantly, while PPARγ decreased significantly. However, after depletion of ID3, irradiation no longer inhibited the expression of PPARγ protein (Fig. 4D). Meanwhile, clonogenic assay also showed that PPARγ inhibitor T0070907 could significantly enhance the cell survival rate of HCT116 after irradiation, but the effect of T0070907 on the cell survival rate of HCT116-ID3 KD after irradiation was significantly reduced (Fig. 4E, F). Similarly, comet assay also indicated that T0070907 could make more residual DSBs in HCT116 cells, but was no longer effective in ID3-depleted HCTl16 cells after 6-Gy irradiation (Fig. 4G). These results suggest that PPARγ and ID3 form a positive feedback loop and jointly affect the radiosensitivity of colorectal cancer cells, and ID3 plays a crucial node role in PPARγ that enhances the radiosensitivity of colorectal cancer cells.

### In vivo experiments verified the effect of ID3 on the radiosensitivity of colorectal cancer

**Fig. 5:**
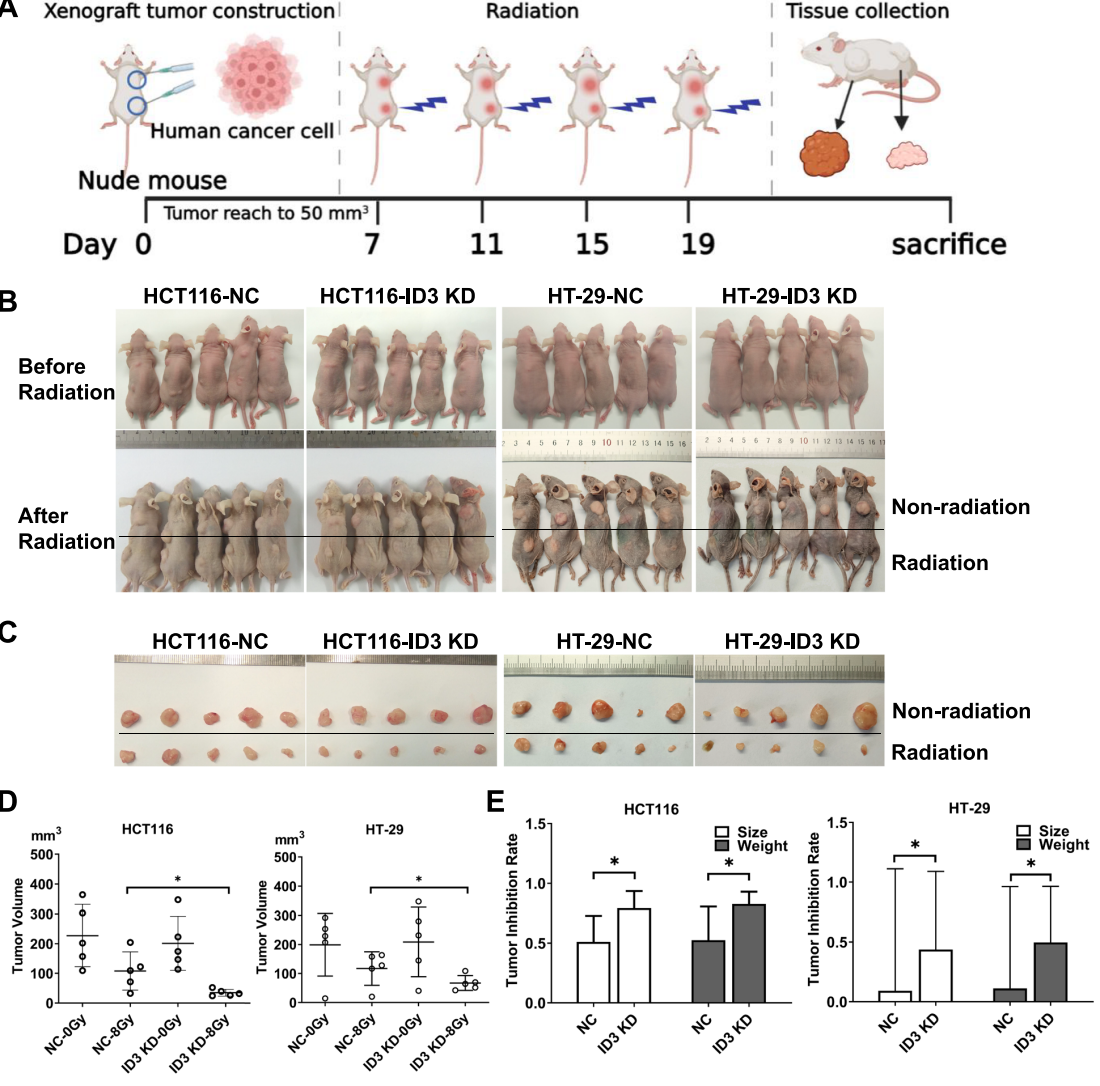
Animal experiments verified the effect of ID3 on the radiosensitivity of HCT116 and HT-29 cells. **A** The experimental scheme for xenograft tumor irradiation. **B** Images of xenograft tumors in nude mice before and after irradiation. **C** Images of xenograft tumors stripped from nude mice. The tumor volume was measured (**D**) and the tumor inhibition rate was analyzed (**E**). Data are expressed as mean ± SD (*n *= 5). *P < 0.05. NC: siRNA control, PC: pcDNA3.1 control, KD: knockdown, OE: overexpress

To further verify the effect of ID3 on the radiosensitivity of colorectal cancer cells, we constructed subcutaneous xenograft tumor models of HCT116 and HT-29 cells in nude mice. The xenograft tumors were irradiated with X-rays when they reached 50 mm^3^ in size to observe the inhibitory effect of irradiation (Fig. 5A). The results showed that after irradiation, the hind leg xenograft tumors in the NC and ID3 KD groups showed different degrees of growth inhibition compared with the non-irradiated foreleg tumors (Fig. 5B and C). However, by comparing the inhibition rate, the inhibition of the Id3 KD group was more obvious regardless of tumor volume or tumor weight (Fig. 5D and E). Colorectal cancer cells with low expression of ID3 were more sensitive to irradiation in mice.

### Retrospective analysis of the relationship between ID3 and radiotherapy efficacy of rectal cancer

**Fig. 6:**
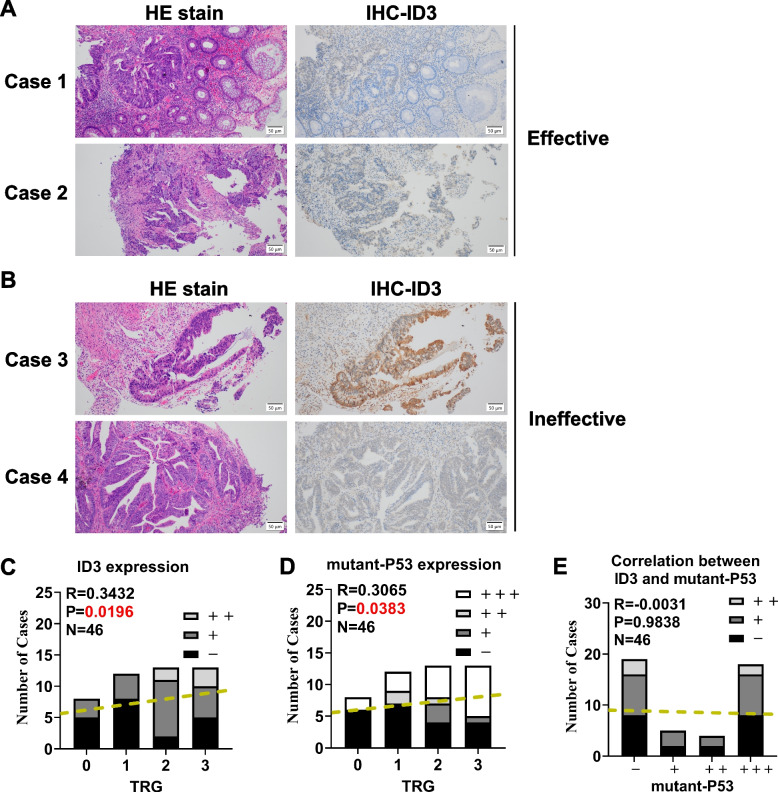
Retrospective analysis of the relationship between ID3 and radiotherapy efficacy of rectal cancer. Representative HE staining and corresponding immunohistochemistry of ID3 in the effective (**A**) and ineffective groups (**B**) are shown. **C** Correlation between the TRG grade and ID3 expression. **D** Correlation between the TRG grade and mutant p53 expression. **E** Correlation between ID3 expression and mutant p53 expression

Of the 46 patients, 20 patients were defined as good responders (TRG 0–1) and 26 patients as poor responders (TRG 2–3) (Tables 1 and 2). ID3 expression in the paraffin specimens of 46 rectal cancer patients without any treatment was detected by immunohistochemistry (Fig. 6A and B). The results showed that there were 7 cases with ID3 + expression and 13 cases with ID3 − expression in the effective radiotherapy group. In the ineffective radiotherapy group, ID3 +  + was expressed in 5 cases, ID3 + in 14 cases and ID3 − in 7 cases. Statistical analysis (Fig. 6C and Table 2) showed that the expression composition ratio of ID3 had a statistically significant difference in the response to radiotherapy and chemotherapy (*P* = 0.0196). Thus, the expression of ID3 may affect the efficacy of radiotherapy in patients with rectal cancer and could be used as an indicator in individualized radiotherapy.

**Table 2:** Correlation analysis between the TRG grade, ID3 and mutant p53 expression

Case number	TRG grade				*P* value
	Grade 0	Grade 1	Grade 2	Grade 3	
ID3 expression					0.0196*
-	5	8	2	5	
+	3	4	9	5	
+ +	0	0	2	3	
p53 expression					0.0383*
-	6	7	4	4	
+	0	0	3	1	
+ +	0	2	1	0	
+ + +	2	3	5	8	

^*^ *P* < 0.05

However, it is still unclear whether the good responders had early-stage cancers or advanced cancers, and similarly, whether the poor responders had early or advanced cancers. Therefore, it would be helpful to investigate the expression of ID3 in early and advanced tumors to understand the relationship between ID3 expression and tumor response. To determine whether TRG or ID3 expression varies between early and advanced tumors, we analyzed the correlation between TRG and clinical stage as well as the association between ID3 expression and clinical stage. It is interesting to note that there was a significant correlation between TRG and the clinical stage, while there was no correlation between ID3 expression and the clinical stage (Table 3).

**Table 3:** Correlation analysis between the TRG, ID3 and clinical stage

Case number	Clinical stage		*P* value
	Early cancer (I-II)	Advanced cancer (III-IV)	
TRG			0.0205*
0–1	8	18	
2–3	12	8	
ID3 expression			0.7359
-	9	11	
+	9	12	
+ +	2	3	

^*^ *P* < 0.05

In addition, we analyzed the immunohistochemical results of mutant p53 from the clinical data of patients with rectal cancer. Similar to the studies of the same kind [18], rectal cancer patients harboring p53 mutations showed a reduced sensitivity compared to patients lacking p53 or those with wild–type p53 (Fig. 6D and Table 2). However, there is a lack of correlation between p53 and ID3, which is the same as our cytological results (Fig. 6E). In addition, among 23 patients with mutant p53 +  + or +  +  + , 7 patients with TRG grade 0–1 were effective and 14 patients with TRG grade 2–3 were ineffective in radiotherapy. In 14 cases of ineffective group, only 4 cases (28.6%) with ID3 negative, while in 7 cases of effective group, 5 cases (71.4%) were ID3 negative. The effective rate of radiotherapy in patients with mutant p53 negative is only 28.3%, but in patients with mutant p53 negative or ID3 negative, the effective rate can reach to 43.5%. These results indicated that ID3 on the radiosensitivity of rectal cancer was through p53–independent pathway. And for rectal cancer patients with p53 mutations, the low expression of ID3 may be one of the indicators of radiotherapy benefit.

## Discussion

Many studies including the current study concluded that NCRT might bring long-term survival benefits to patients with locally advanced rectal cancer, especially those at high risk [19]. However, different tumors or the same tumor type in different individuals have differing sensitivity to radiotherapy because of the different gene profiles. Much progress has been made in basic research and clinical practice of tumor tolerance to radiotherapy [20, 21]. However, because of the heterogeneity of tumors, the issue of tumor insensitivity to radiotherapy has not been completely solved, especially in the treatment of rectal cancer. Tumor radiosensitivity is regulated by various genes, including DNA damage repair-related genes, apoptosis-related genes, cell hypoxia-related genes, cell cycle-related genes, and cell stemness-related genes [22–24].

In this study, we analyzed the effect of ID3 on the radiosensitivity of HCT116 and HT-29 cell lines by clonogenic and cell proliferation assays. When exposed to 2–8 Gy irradiation, the radiosensitivity of both HCT116 and HT-29 cell lines increased with the decrease in ID3 expression. *Tp53* is a crucial gene in many kinds of tumors [25] that is directly involved in the process of DNA damage repair and enhances the radiosensitivity of rectal cancer [26, 27]. Indeed, reintroducing a functional p53 alone has been shown to robustly induce tumor regression. In addition, an active p53 pathway is essential for effective radiotherapy. The emerging cyclotherapy, in which p53 acts as a chemoprotector for normal tissues, further expands the usefulness of p53 activators [28]. However, P53 is degraded by ubiquitination of various molecules including MDM2, and p53 cannot maintain high expression for a long time even by gene therapy [29]. So far, none have been approved by the FDA. The radiosensitivity of HCT116 with wild–type p53 and HT-29 with mutant p53 changed when ID3 expression changed, indicating that the effect of ID3 on the radiosensitivity of rectal cancer was not regulated by p53. For rectal cancer patients with p53 mutation or deletion but low expression of ID3, it is possible to obtain a better therapeutic effect of NCRT.

DNA damage caused by DSBs is the most direct reason for cancer cell death caused by irradiation [30]. The ability of cells to correctly detect and repair DSBs is very important to maintain genomic stability. However, for radiotherapy, the weaker the repair ability of DSBs, the better the effect of radiotherapy. Augmented DSB repair capacity is a major cause of radio- and chemoresistance and, ultimately, cancer recurrence [31]. When DSBs occur, it initiates a signaling cascade that begins with the phosphorylation of histone variant H2AX (γH2AX) at the DSB site, followed by the recruitment of upstream factors, including MDC1 [32]. γH2AX expression is an early cellular response to the induction of DSBs, and its detection has become a highly specific and sensitive molecular marker to monitor the initiation and resolution of DNA damage [33]. MDC1 amplifies DNA damage signals by binding to γH2AX and then binding at DNA damage sites and retaining additional DNA damage response factors [34]. It is generally believed that the accumulation of these factors at DSB sites contributes to DNA damage repair and checkpoint control [31]. Therefore, MDC1 has been recognized as the "main regulator" to regulate the specific chromatin microenvironment required to maintain genomic stability [35]. We found that when colorectal cancer cells were exposed to irradiation, ID3, γH2AX, and MDC1 increased and formed foci to repair DNA damage caused by irradiation. However, when ID3 was decreased, only MDC1 expression decreased, but γH2AX did not change. Both co-IP and immunofluorescence co-localization assays showed that ID3 formed a complex with MDC1 to regulate the ability of DNA damage repair. Recent research [35] demonstrated that ID3 is very important for stabilizing the combination of MDC1 and γH2AX. Eliminating the interactions between MDC and γH2AX will destroy the formation of MDC1 foci induced by irradiation and make cells sensitive to irradiation [35]. Thus, ID3 is very important for the radiosensitivity of colorectal cancer cells.

Our data also emphasized the regulatory positive feedback relationship between ID3 and PPARγ. PPARγ is a ligand-dependent transcription factor that belongs to the type II nuclear hormone receptor superfamily [36]. PPARγ can inhibit the growth of malignant tumors by affecting cell proliferation, apoptosis, angiogenesis, inflammation, and metastasis [37]. In addition, PPARγ has been proven to enhance the radiosensitivity of cancer cells, and agonists of PPARγ have been demonstrated to affect the radiosensitivity of various cancers [38, 39]. Our data showed that ID3 was negatively correlated with PPARγ. When ID3 increased it inhibited PPARγ. Once PPARγ was inhibited by ID3, it further promoted ID3 and formed a positive feedback loop to gradually enhance the malignancy of colorectal cancer cells and radiotherapy resistance. Further research showed that the regulation of PPARγ in radiosensitivity needs to be reflected by ID3. Thus, the role of ID3 in the radiosensitivity of colorectal cancer has been further highlighted in this study. However, how ID3 and PPARγ regulate each other needs further investigation.

Our study also verified the role of ID3 in the radiosensitivity of colorectal cancer in vivo. In addition to the TNM stage after surgery, the TRG of postoperative pathology should also be considered in the evaluation of radiotherapy efficacy. TRG has prognostic value in patients with locally advanced rectal cancer undergoing preoperative radiotherapy and chemotherapy. According to previous data, TRG is closely related to the improvement of metastasis-free and disease-free survival after preoperative neoadjuvant chemoradiotherapy [40]. Although many TRG models have been proposed, the four-tier AJCC rectal cancer TRG system has been shown to be more accurate than other systems and was therefore chosen for this study [14]. Our xenograft tumor irradiation model in nude mice and the retrospective analysis of rectal cancer patients undergoing NCRT supported the view that ID3 can affect the radiosensitivity of colorectal cancer.

**Fig. 7:**
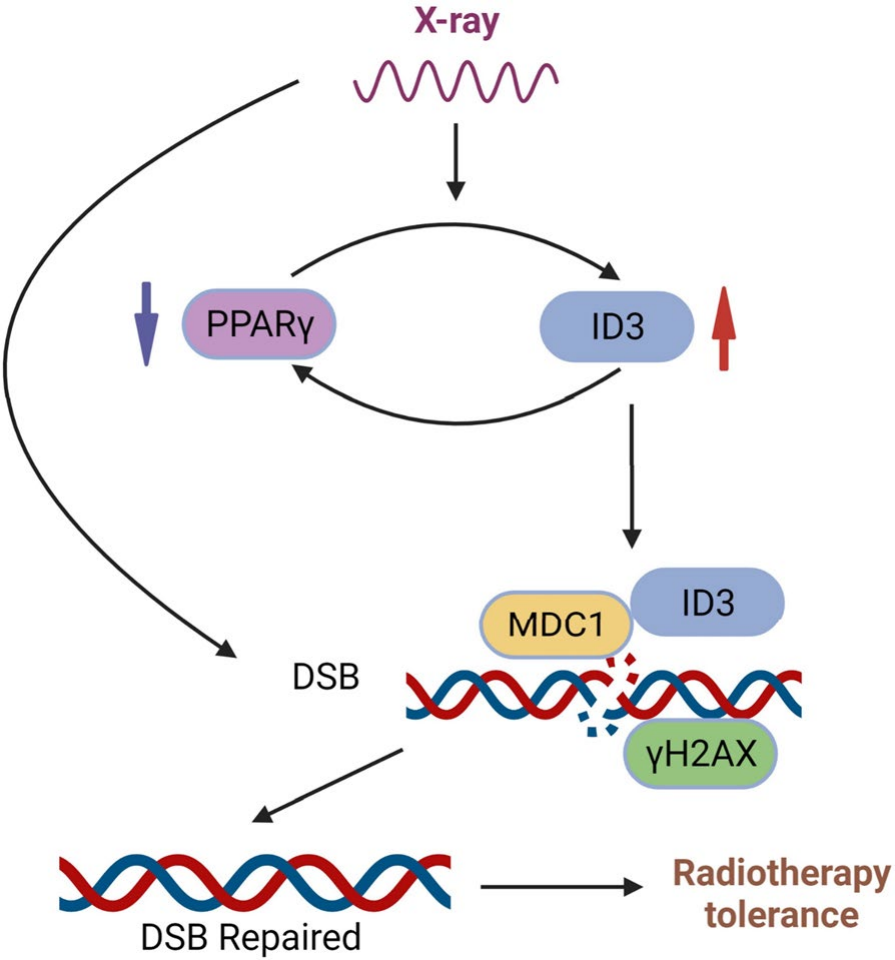
Schematic illustration of ID3 and PPARγ forming a positive feedback loop to promote the repair of DNA damage and affecting the radiosensitivity of colorectal cancer cells

In conclusion, through communication with MDC1, ID3 and PPARγ formed a positive feedback loop to promote the repair of DNA damage, thus affecting the radiosensitivity of colorectal cancer cells (Fig. 7). Preoperative neoadjuvant chemoradiotherapy for rectal cancer patients with low expression of ID3 can obtain a better curative effect, regardless of *Tp53* gene status.

## Supplementary Information

**Additional file 1: Figure S1.** Original picture of Fig. 1A. **Figure S2.** Original picture of Fig. 2A and B. **Figure S3.** Original picture of Fig. 3A, E and F. **Figure S4.** Original picture of Fig. 4A, B, C and D.

## Data Availability

The datasets used and/or analyzed during the current study are available from the corresponding author on reasonable request.

## Abbreviations

ID3: Inhibitor of differentiation 3
NCRT: Neoadjuvant chemoradiotherapy
bHLH: Basic helix–loop–helix
TRG: Tumor regression grade
PPARγ: Peroxisome Proliferator-Activated Receptor γ
MDC1: Mediator of DNA damage checkpoint 1
IR: Irradiation
DSB: Double-strand break
