# Supplementary material for: A positive feedback loop between ID3 and PPARγ via DNA damage repair regulates the efficacy of radiotherapy for rectal cancer
Source: BMC Cancer. 2023 May 11;23:429. doi: 10.1186/s12885-023-10874-7 (PMC10176823; doi:10.1186/s12885-023-10874-7)
Supplement: Supplementary file 1 — Additional file 1: Figure S1. Original picture of Fig. 1A. Figure S2. Original picture of Fig. 2A and B. Figure S3. Original picture of Fig. 3A, E and F. Figure S4. Original picture of Fig. 4A, B, C and D. [file 12885_2023_10874_MOESM1_ESM.pdf]

A

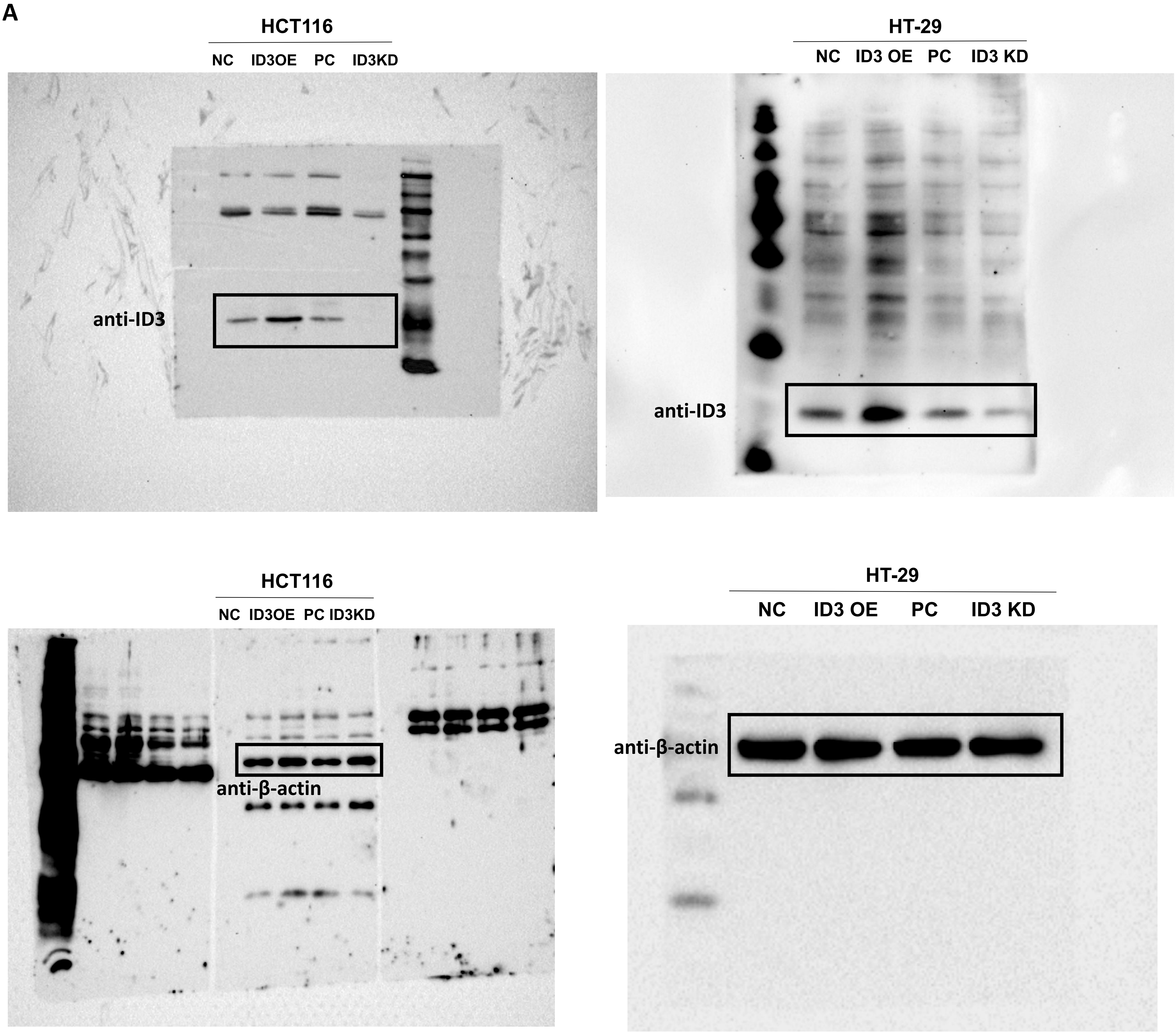

Figure S1 Original picture of figure 1A. Western blotting confirmed ID3 expression in colorectal cancer cells.

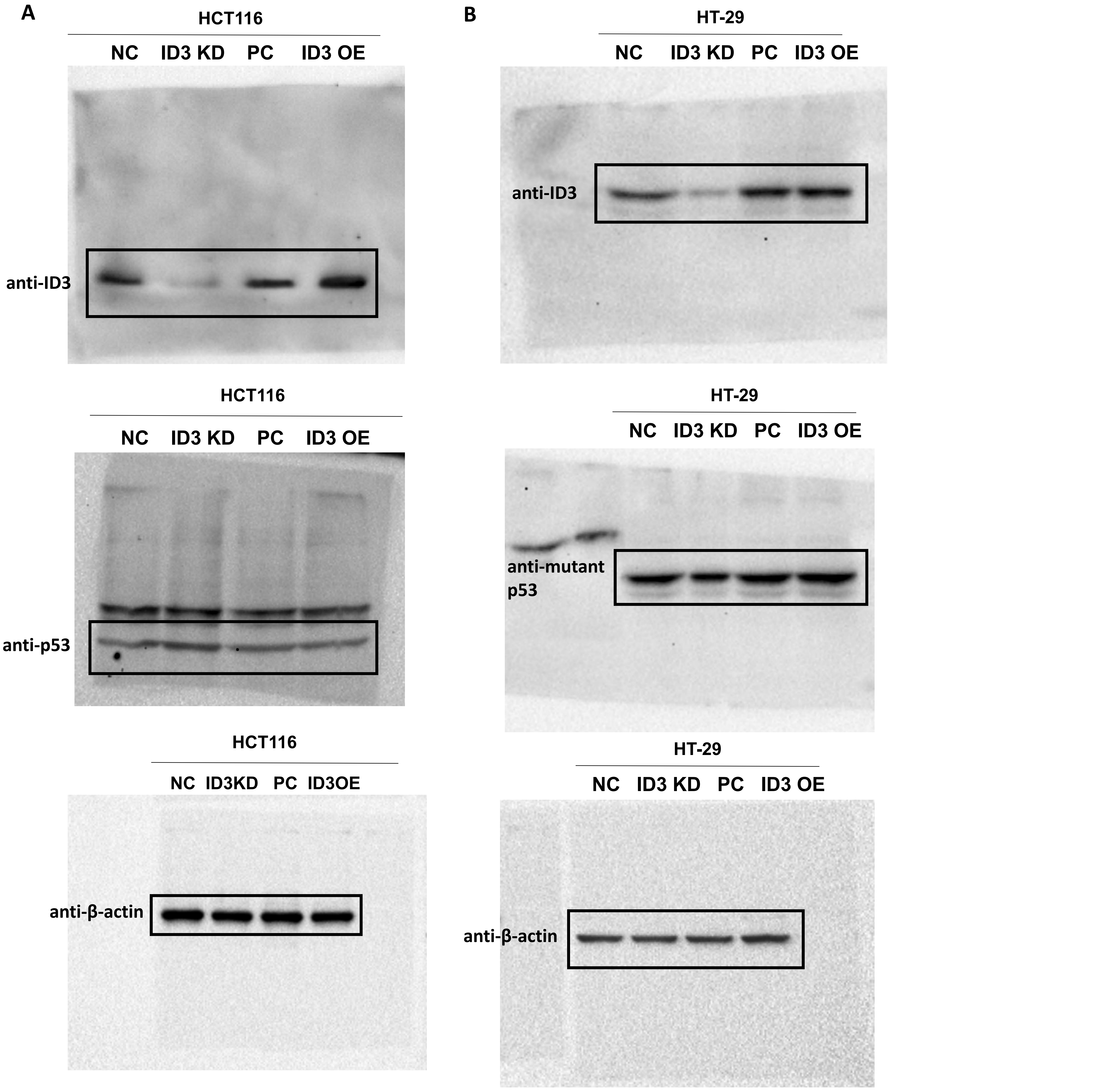

Figure S2 Original picture of figure 2A and 2B.

A. Western blotting confirmed ID3 and p53 expression in HCT116 cells.

B. Western blotting confirmed there was no correlation between ID3 and mutant p53 expression in HT-29 cells.

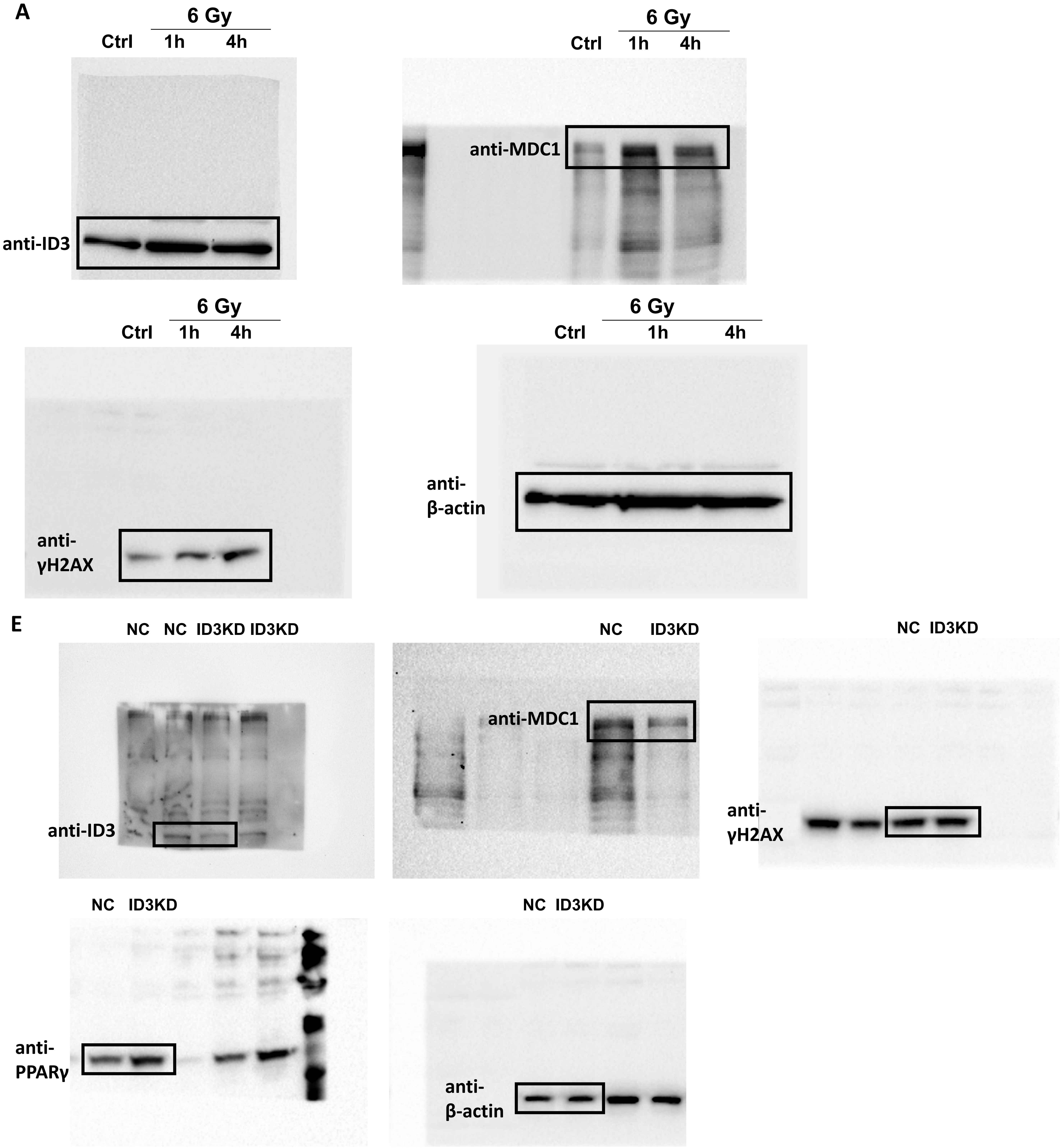

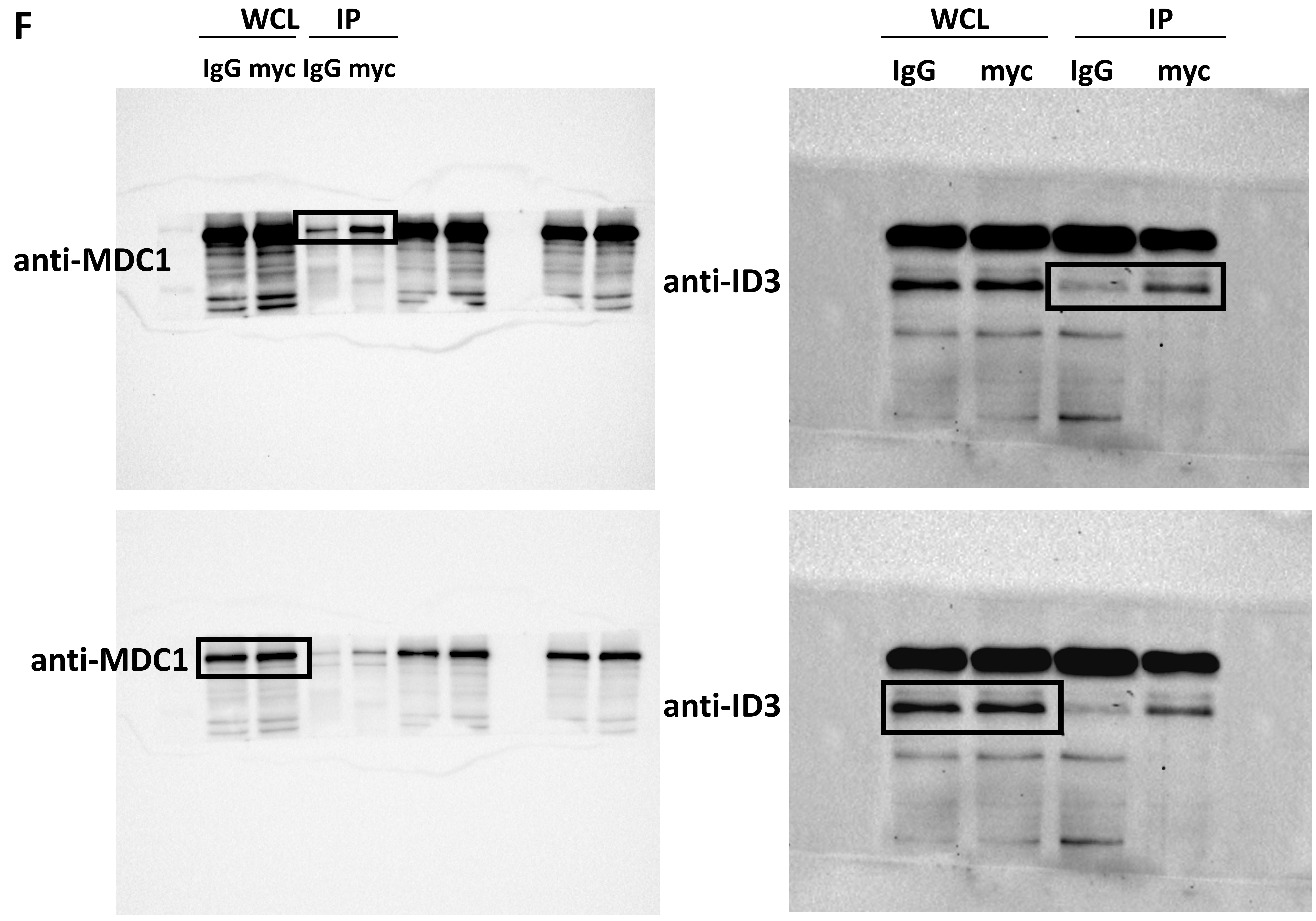

Figure S3 Original picture of figure 3A, 3E and 3F.
A. Western blotting demonstrated the expression of ID3, MDC1 and γH2AX.
E. Western blotting confirmed the expression of MDC1, γH2AX and PPARγ accompanied by ID3 knockdown.
F. Lysates of HCT116 cells were subjected to co-immunoprecipitation using an anti-Myc antibody followed by western blotting using anti-MDC1 and anti-ID3 antibodies.

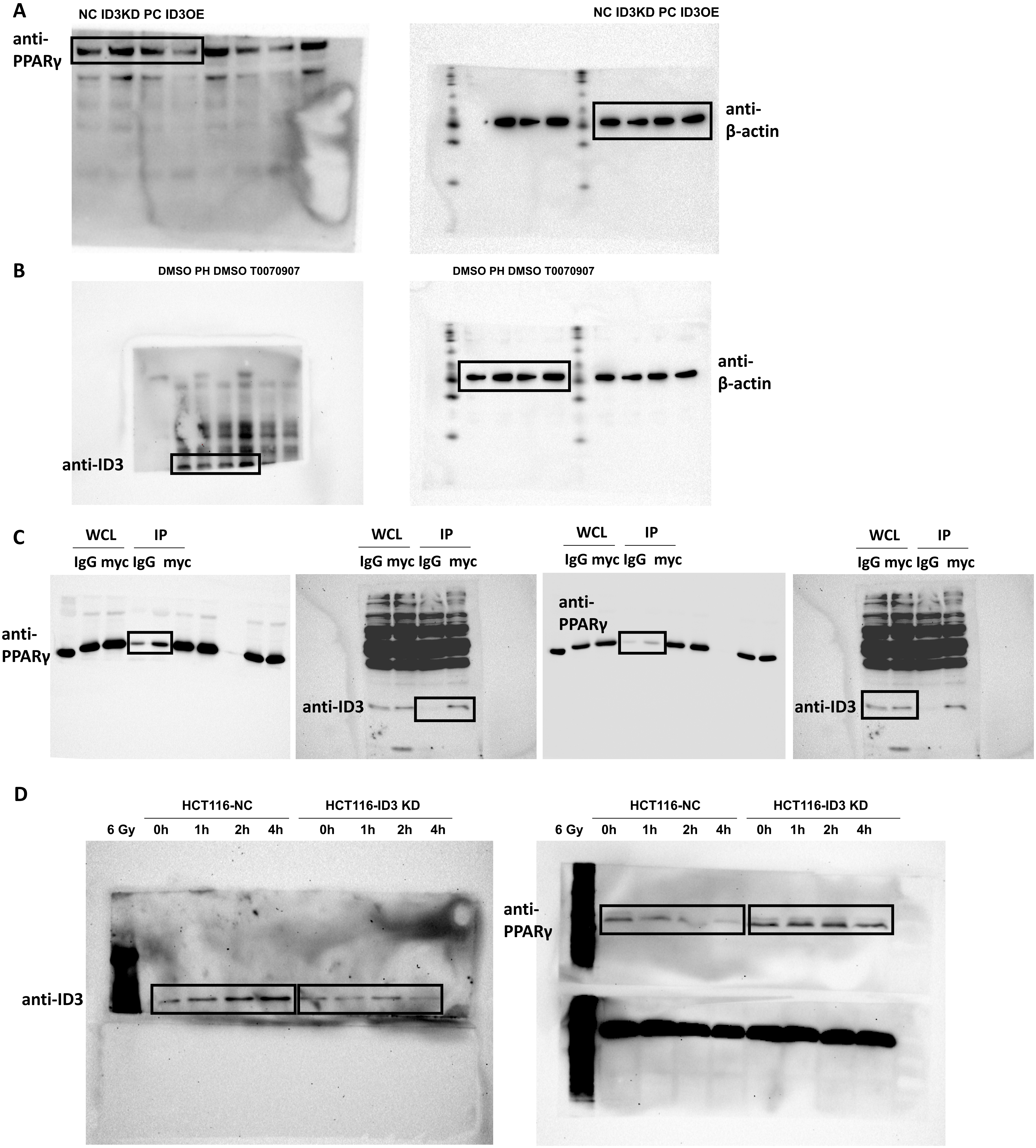

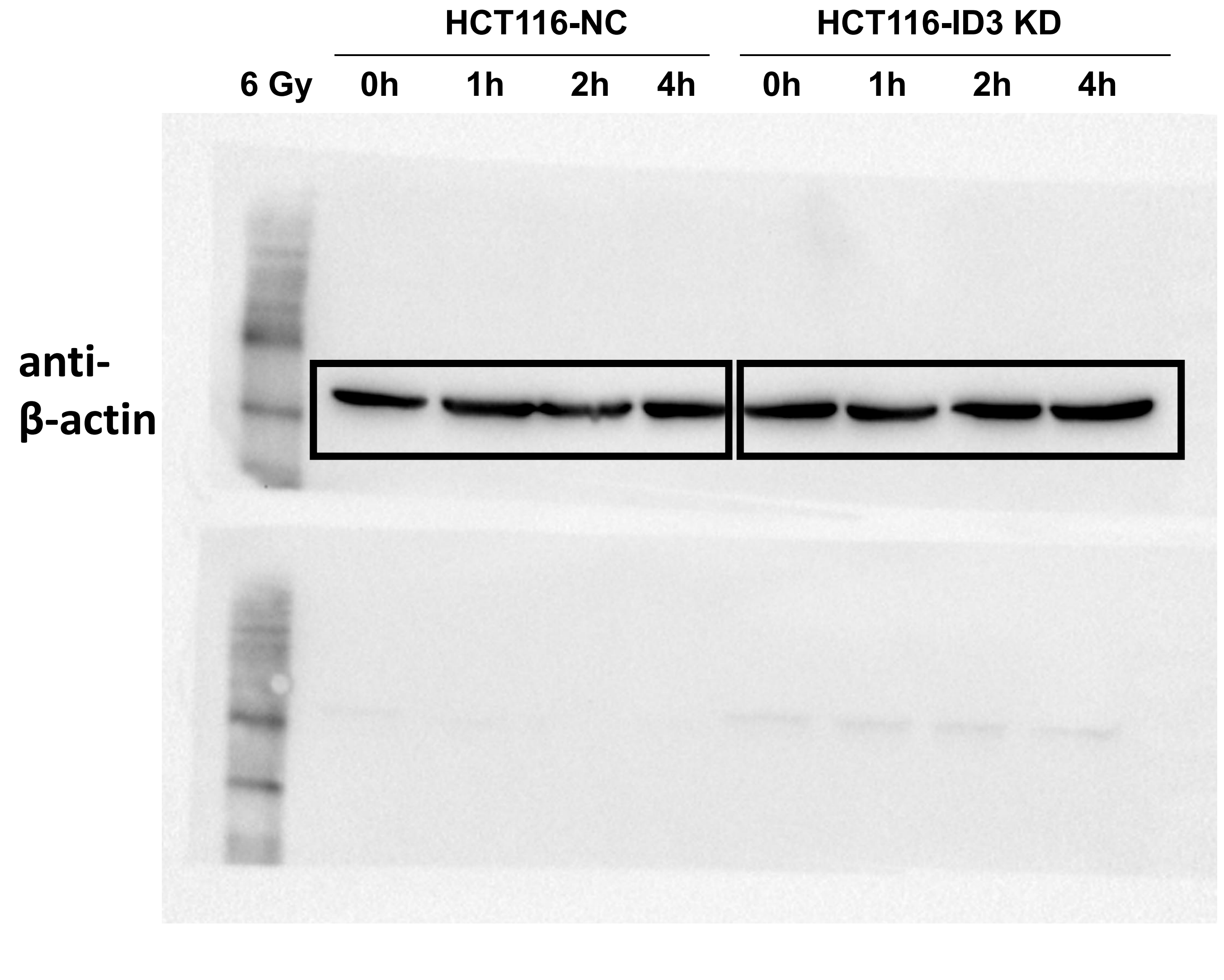

Figure S4 Original picture of figure 3A, 3B, 3C and 3D.
A. Western blotting confirmed PPARγ expression in HCT116 cells with ID3 knockdown or overexpression.
B. Western blotting confirmed ID3 expression in HCT116 cells treated with PPARγ agonist pioglitazone hydrochloride (PH) or PPARγ inhibitor T0070907.
C. Lysates of HCT116 cells were subjected to co-immunoprecipitation using an anti-Myc antibody followed by western blotting using anti-PPARγ and anti-ID3 antibodies.
D. Western blotting confirmed ID3 and PPARγ expression in HCT116-NC and HCT116-Id3 KD cells after irradiation.
